# A case of acute tubulointerstitial nephritis with suspected Saikokaryukotsuboreito involvement responding to glucocorticoid therapy

**DOI:** 10.1007/s13730-025-01012-2

**Published:** 2025-06-26

**Authors:** Shun Ishibashi, Toshiaki Usui, Masaki Baba, Tsuyoshi Tsukada, Kohei Inoue, Shintaro Furuno, Masahiro Niisaka, Soichiro Nomura, Takuya Harada, Tomoki Akiyama, Ryota Ishii, Ryoya Tsunoda, Akiko Fujita, Hirayasu Kai, Naoki Morito, Kaori Mase, Chie Saito, Joichi Usui, Motohiro Sekiya, Kunihiro Yamagata

**Affiliations:** 1 Department of Nephrology, Institute of Medicine, University of Tsukuba, 1-1-1 Tennodai, Tsukuba, Ibaraki 305-8575 Japan; 2 Doctoral Program in Medical Sciences, Graduate School of Comprehensive Human Sciences, University of Tsukuba, 1-1-1 Tennodai, Tsukuba, Ibaraki 305-8575 Japan; 3 Diagnostic Pathology, Institute of Medicine, University of Tsukuba, 1-1-1 Tennodai, Tsukuba, Ibaraki 305-8575 Japan; 4 Department of Internal Medicine, Ibaraki Western Medical Center, 555 Otsuka, Chikusei, Ibaraki 308-0813 Japan; 5 Department of Internal Medicine, Moriya Keiyu Hospital, 980-1 Tatsuzawa, Moriya, Ibaraki 302-0118 Japan; 6 Department of Nephrology, Hitachi General Hospital, 2-1-1 Jonancho, Hitachi, Ibaraki 317-0077 Japan; 7 Department of Nephrology, Tsukuba Gakuen Hospital, 2573-1 Kamiyokoba, , Tsukuba, Ibaraki 305-0854 Japan; 8 Department of Nephrology, Ibaraki Prefectural Central Hospital, 6528 Koibuchi, Kasama, Ibaraki 309-1793 Japan; 9 Department of Nephrology, Ibaraki Clinical Education and Training Center, University of Tsukuba Hospital, 6528 Koibuchi, Kasama, Ibaraki 309-1793 Japan; 10 Department of Endocrinology and Metabolism, Institute of Medicine, University of Tsukuba, 1-1-1 Tennodai, Tsukuba, Ibaraki 305-8575 Japan

**Keywords:** Acute tubulointerstitial nephritis (AIN), Saikokaryukotsuboreito, Drug-induced kidney injury, Glucocorticoid therapy

## Abstract

Drug-induced kidney injury is a major cause of acute tubulointerstitial nephritis (AIN). The standard treatment for AIN is discontinuation of the causative agent; however, if discontinuation alone is insufficient, glucocorticoid therapy may be effective. A 51-year-old woman was admitted to our hospital with acute kidney injury, presenting with an estimated glomerular filtration rate (eGFR) of 6.3 mL/min/1.73m^2^. She was diagnosed with AIN after a renal biopsy. Drug-induced lymphocyte stimulation tests were performed on the herbal medicine she had been taking for 5 months, Saikokaryukotsuboreito (comprising Bupleurum root, Pinellia tuber, Scutellaria root, and Ginseng as key ingredients). The test results were positive for all four components. The patient discontinued the causative agent, and her renal function improved with oral glucocorticoid therapy (prednisolone 30 mg/day). After 6 months of treatment, her renal function stabilized, with only minor residual tubular acidosis. This case of AIN induced by Saikokaryukotsuboreito, which responded to glucocorticoid therapy, highlights the potential for recovery with appropriate treatment.

**Supplementary Information:**

The online version contains supplementary material available at 10.1007/s13730-025-01012-2.

## Introduction

Herbal medicines, including Chinese herbal medicine and traditional Japanese herbal medicine, are covered by insurance in Japan and are clinically used to improve symptoms that cannot be addressed by Western medicine alone. While certain Chinese herbal medicines are known to cause aristolochic acid nephropathy (AAN), which leads to renal interstitial injury [1, 2], the nephrotoxicity of other herbal medicines is less well understood [3]. Saikokaryukotsuboreito (Chai-Hu-Jia-Long-Gu-Mu-Li-Tang in Chinese) is a traditional Japanese herbal medicine that is used clinically to treat irritable mental symptoms [4].

We herein report the successful treatment of a patient with acute tubulointerstitial nephritis (AIN) induced by Saikokaryukotsuboreito, who had extensive acute tubulitis on renal biopsy. Drug-induced lymphocyte stimulation tests (DLST) were performed on Saikokaryukotsuboreito, which contains Bupleurum root, Pinellia tuber, Scutellaria root, and Ginseng as its key ingredients. Positive results were recorded for all four components. The patient’s renal function did not improve with the discontinuation of the causative agent alone but improved following oral glucocorticoid therapy.

## Case report

A 51-year-old woman was diagnosed with diabetes mellitus 10 years prior to presentation, for which she has been treated as an outpatient in the Department of Endocrinology and Metabolism at our hospital. Five months prior to her presentation at the hospital, her estimated glomerular filtration rate (eGFR) was 62.1 mL/min/1.73 m^2^. Around the same time, she began self-medicating by taking Saikokaryukotsuboreito twice a week. This herbal medicine, which does not contain aristolochic acid (a known nephrotoxin associated with Chinese herb nephropathy), had been prescribed to one of her family members for irritable mental symptoms. It is important to note that the patient herself was not seeing a psychiatrist and did not have anorexia. A month prior to presentation, she developed nausea, loss of appetite, and back pain. She visited a general practitioner, who diagnosed gastroenteritis and prescribed domperidone, sodium alginate, vonoprazan fumarate, and acetaminophen; however, her symptoms did not improve. Blood analyses revealed severe renal dysfunction with an eGFR 8.2 mL/min/1.73 m^2^, resulting in immediate admission to our hospital. Upon admission, her physical examination revealed: height 159.6 cm, weight 50.4 kg, blood pressure 118/65 mmHg, pulse rate 86 beats per minute, and body temperature 36.6 ℃. Throughout the clinical course, she did not develop fever or skin rash. In the past 6 days, her eGFR deteriorated to 6.3 mL/min/1.73 m^2^. Although urinary protein was 0.42 g/g Cre and microscopic hematuria was absent, she had aseptic pyuria, elevated levels of N-acetyl-β-D-glucosaminidase (NAG) and β2-microglobulin (β2MG) in the urine, and eosinophiluria (Table 1). Bilateral renal enlargement was observed on computed tomography. While all newly initiated drugs suspected of causing drug-induced renal impairment were halted and intravenous fluids were administered, the patient’s renal function did not improve. We performed a DLST on the suspect drugs. The results showed that of the drugs she had started within the past 6 months, only Saikokaryukotsuboreito had a positive DLST result (see Table 2).

**Table 1:** Laboratory findings on admission

Urinalysis		
pH	5.5	
Protein	0.43	g/gCr
Hematuria	0–1	/high power field
Leukocyturia	10–19	/high power field
Eosinοphiluria	+	
Glucosuria	4 +	
β2-microglobulin	3535	μg/L
β2-microglobulin	4.9	mg/gCr
N-acetyl-β-D-glucosaminidase	9.6	IU/L
N-acetyl-β-D-glucosaminidase	13.3	mIU/gCr
Complete blood count		
White blood cells	11,600	/*µ*L
Neutrophil	79.2	%
Eosinophil	2.2	%
Basophil	0.4	%
Monocytes	4.7	%
Lymphocytes	13.5	%
Red blood cells	4.11 × 10^6^	/*µ*L
Hemoglobin	11.7	g/dL
Hematocrit	35.0	%
Platelets	57.7 × 10^4^	/*µ*L
Coagulation test		
Activated partial thromboplastin time	28.6	sec
Prothrombin time	87.1	%
Biochemistry		
Aspartate aminotransferase	16	U/L
Alanine aminotransferase	9	U/L
Alkaline phosphatase	47	U/L
Lactate dehydrogenase	159	U/L
γ-Glutamyl transpeptidase	20	U/L
Total bilirubin	0.8	mg/dL
Creatine kinase	62	U/L
Albumin	4.1	g/dL
Sodium	134	mEq/L
Potassium	4.4	mEq/L
Cl	100	mEq/L
Blood urea nitrogen	53.0	mg/dL
Creatinine	6.16	mg/dL
Creatinine estimated glomerular Filtration rate	6.3	mL/min/1.73 m^2^
C-reactive protein	2.35	mg/dL
Immunoserological test		
Immunoglobulin G	1538	U/mL
Immunoglobulin A	255	mg/dL
Immunoglobulin M	127	mg/dL
Complement component 3	139	mg/dL
Complement component 4	54	mg/dL
50% hemolytic unit of complement	93.1	U/mL
Antinuclear antibody	< 40	
Proteinase-3 anti-neutrophil cytoplasmic antibody	< 1.0	IU/mL
Myeloperoxidase-anti-neutrophil cytoplasmic antibody	< 1.0	IU/mL

**Table 2:** Drug-induced lymphocyte stimulation tests (DLST)

Drug and component	Positivity(%)^a^	Judgement
Saikokaryukotsuboreito	214	Positive
Bupleurum root	218	Positive
Pinellia tuber	594	Positive
Scutellaria root	454	Positive
Ginseng	487	Positive
Acetaminophen	130	Negative
Sodium alginate	124	Negative
Kakkonto	134	Negative
Sitagliptin	120	Negative
Metformin	115	Negative
Canagliflozin	102	Negative
Metoclopramide	124	Negative
Fenofibrate	121	Negative
Esomeprazole	121	Negative
Vonoprazan	137	Negative

^a^Criteria for positivity: negative, < 179%; pseudo-positive, 180–199%; positive, ≥ 200%

**Fig. 1:**
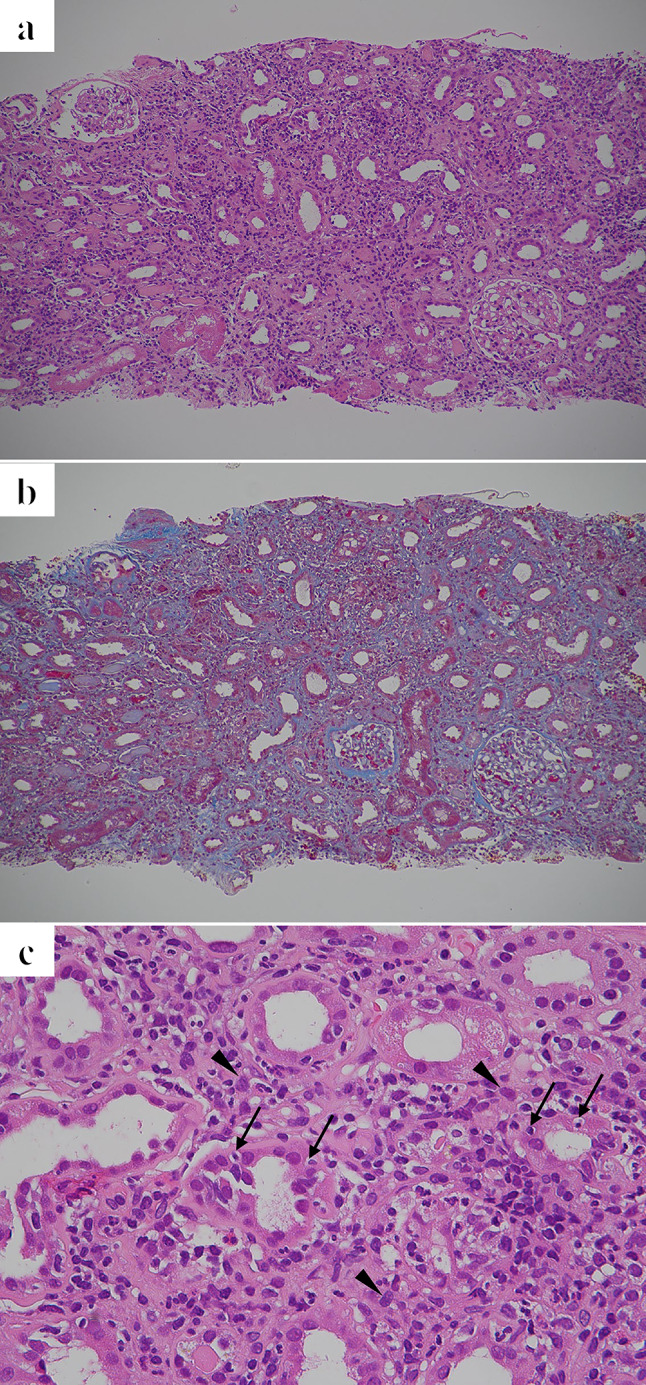
Light microscopic findings. Mononuclear inflammatory cells diffusely infiltrated in the interstitium with tubular atrophy (**a**: hematoxylin–eosin stain, **b**: Masson’s trichrome stain). Mild interstitial fibrosis was also found. Tubulitis was frequently seen (**c**: hematoxylin–eosin stain, arrows). Several lymphocytes (arrow) and giant cell-like cells (arrowhead) were included. Original magnification: **a**, **b** × 100, **c** × 400

**Fig. 2:**
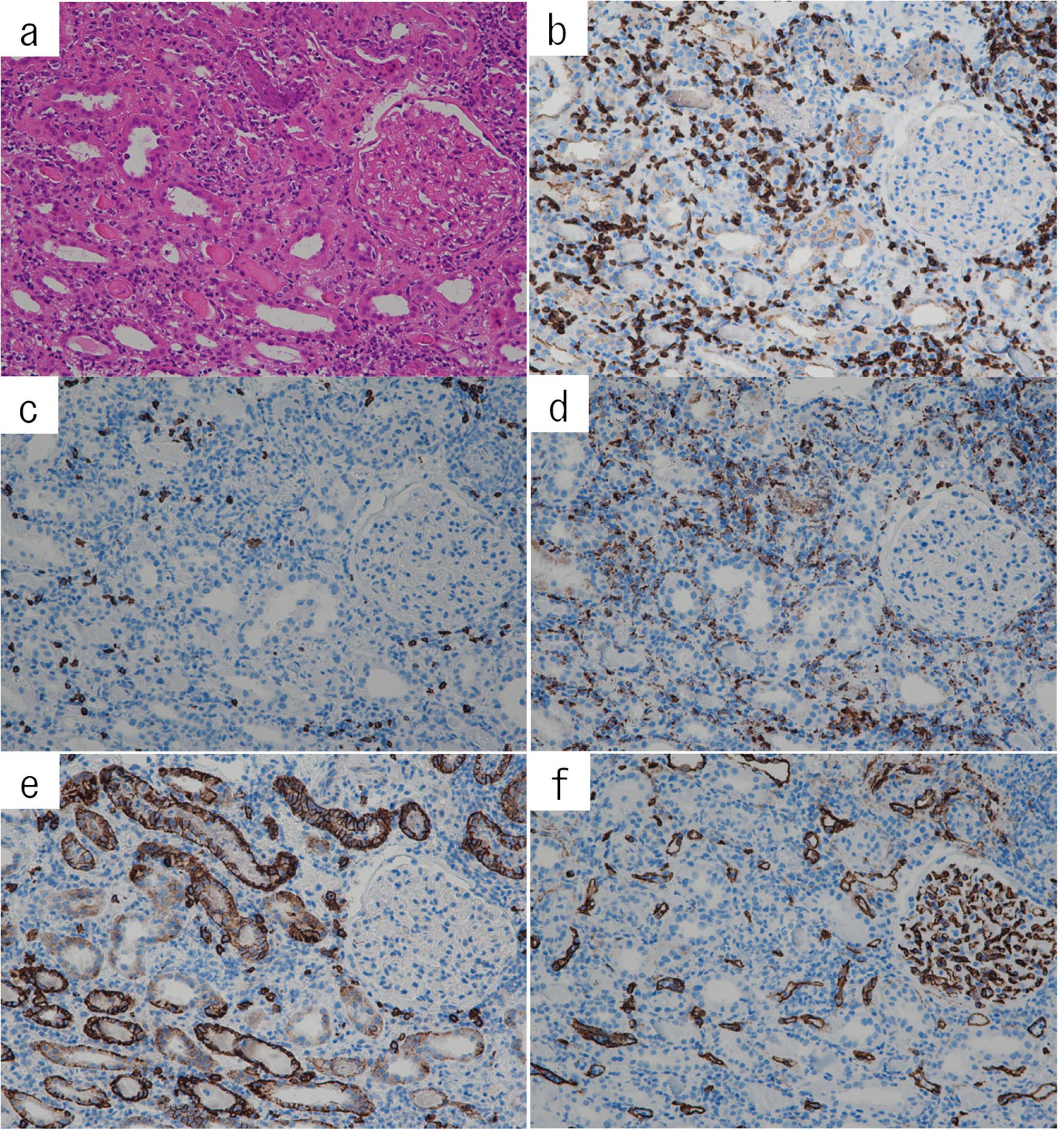
Immunohistochemical analysis of infiltrating inflammatory cells using serial sections. There was diffuse interstitial infiltration (**a**: Hematoxylin–eosin stain). CD3-positive T lymphocytes (**b**) and CD68-positive macrophages (**d**) were mainly infiltrated in the interstitium. CD20-positive B lymphocytes (**c**) and CD138-positive mature plasma cells (**e**) were occasionally found. The structure of peritubular capillaries was preserved, and the inflammatory infiltration intra-peritubular capillaries was scant (f, anti-CD34 stain). Original magnification: **a**–**f**, × 200

On the 15th day of hospitalization, a renal biopsy was performed to confirm the diagnosis. On the light microscopy, twenty-four glomeruli were obtained, with one showing global sclerosis (Fig. 1a, b). Almost all glomeruli showed minor glomerular abnormalities. Abundant mononuclear inflammatory cells diffusely infiltrated in the interstitium with tubular atrophy and mild interstitial fibrosis. Tubulitis was frequently seen (Fig. 1c). Infiltrated eosinophils were rarely found, and interstitial granuloma was not seen. Although there are no typical giant cells, giant cell-like cells are seen, suggesting a foreign body reaction to the suspected drug (Fig. 1c). To determine cell type of infiltration, we analyzed immunohistochemistry (Fig. 2a–f, antibody information; supplemental table). The infiltrate mainly consisted of CD3-positive T lymphocytes and CD68-positive macrophages, while CD20-positive B lymphocytes and CD138-positive mature plasma cells were occasionally found. The structure of peritubular capillaries was preserved, and inflammatory cell infiltration into the peritubular capillaries was scant. Interlobular arteries showed moderate fibrous intimal thickening. Arterioles displayed mild hyaline sclerosis, and no evidence of arteritis was observed. On the immunofluorescence, anti-IgG, IgA, IgM, C3, C1q stain was negative in glomeruli, tubuli, and tubular basement membrane (Sup.1). The pathological diagnosis was acute and chronic tubulointerstitial nephritis without diabetic nephropathy, with no evidence of diabetic nephropathy on histological examination.

Based on the DLST results and renal biopsy pathology, we diagnosed drug-induced acute interstitial nephritis (DI-AIN) due to Saikokaryukotsuboreito exposure, given that the DLST for each component of this herbal medicine (namely Bupleurum root, Pinellia tuber, Scutellaria root, and Ginseng) was positive.

**Fig. 3:**
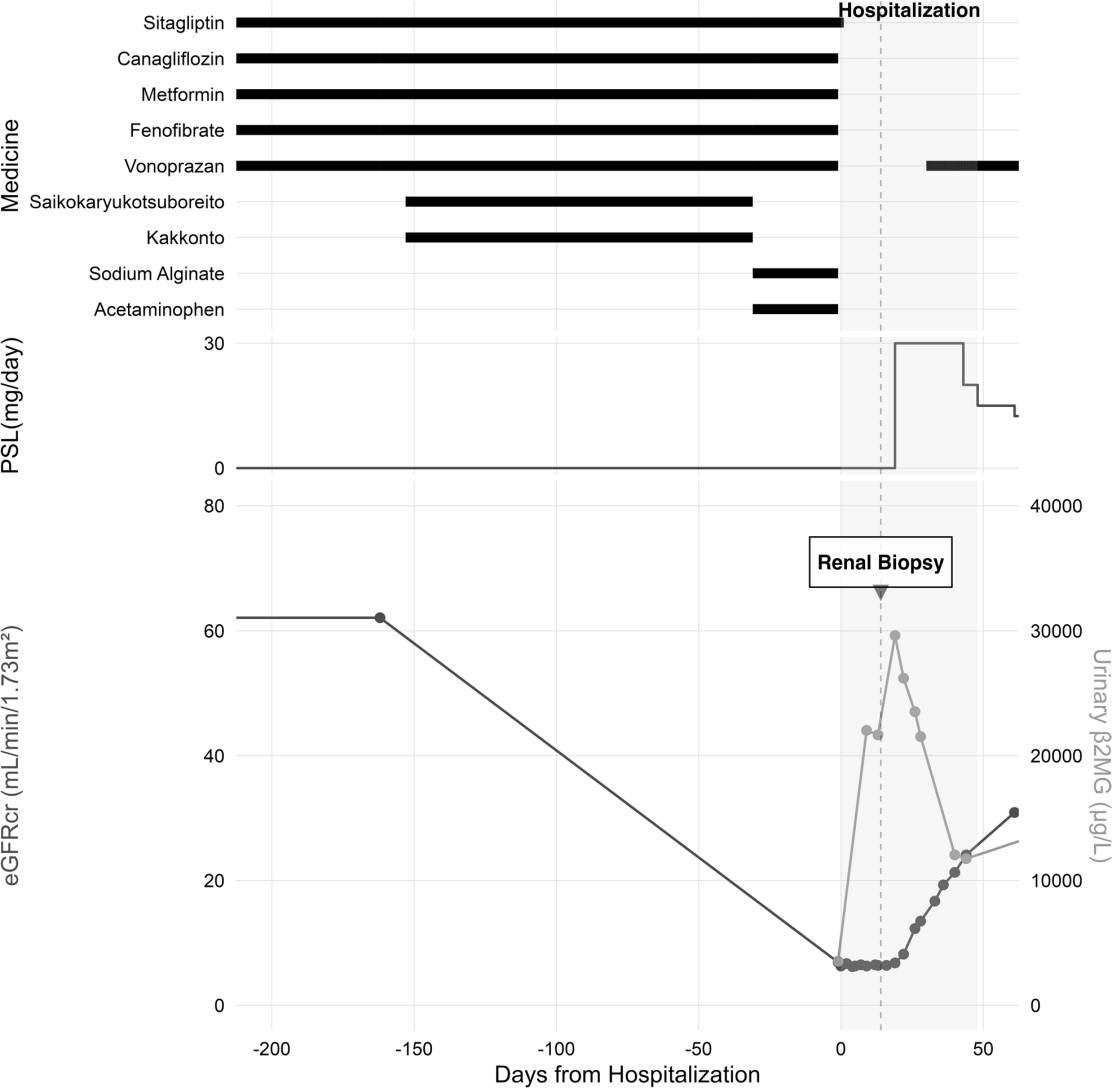
Clinical course of the patient with drug-induced AIN. The upper panel shows the medication history, including chronic medications (sitagliptin, canagliflozin, metformin, fenofibrate, vonoprazan) and recently added drugs (Saikokaryukotsuboreito, Kakkonto, sodium alginate, acetaminophen). The middle panel shows the prednisolone (PSL) treatment starting at 30 mg/day, followed by tapering. The lower panel demonstrates changes in renal function: the black line represents the decline in eGFRcr from baseline and the improvement after PSL initiation, while the gray line represents urinary β2MG levels. The gray shaded area indicates the hospitalization period, with the renal biopsy performed on day 15. Abbreviations: PSL, prednisolone; eGFRcr, estimated glomerular filtration rate based on creatinine; β2MG, β2-microglobulin

On the 21st day of hospitalization, the patient was started on prednisolone (PSL) at a dose of 30 mg/day (0.6 mg/kg/day). After starting PSL, the inflammatory reaction resolved, and renal function improved. By day 45, her eGFR had improved to 24.1 mL/min/1.73 m^2^, and urinary NAG and β2MG concentrations also decreased. She was discharged on the 49th day. The dose of PSL was gradually reduced through outpatient treatment, and 6 months later, PSL administration was terminated. Although she developed tubular acidosis as a sequela of AIN, no recurrence of AIN was observed after PSL discontinuation (Fig. 3).

## Discussion

Drug-induced kidney injury (DKI) is a major cause of acute interstitial nephritis (AIN), with drug-induced AIN (DI-AIN) accounting for approximately 70% of AIN cases [5]. This case highlights, for the first time, that Saikokaryukotsuboreito can induce AIN through an immune-mediated mechanism, similar to other well-characterized drug-induced hypersensitivity reactions. Importantly, although glucocorticoid treatment was not initiated immediately, our patient showed substantial improvement, demonstrating that DI-AIN due to herbal medicines can be effectively diagnosed and treated using established approaches for conventional drug-induced nephropathy.

The diagnosis of Saikokaryukotsuboreito-induced AIN was supported by a comprehensive evaluation. Our patient presented with severe acute kidney injury with minimal urinary abnormalities. Renal biopsy revealed key diagnostic features of drug hypersensitivity: diffuse interstitial inflammation with predominant T lymphocytes and macrophages infiltration, marked tubulitis, and preserved glomerular architecture. This cellular composition is consistent with a cell-mediated immune response characteristic of drug hypersensitivity reactions [6]. The absence of granulomas, giant cells, or immune complex deposits helped exclude other type of AIN [7]. The immunological basis of this reaction was confirmed by positive drug-induced lymphocyte stimulation test (DLST) results for Saikokaryukotsuboreito and all four of its major components (Bupleurum root, Pinellia tuber, Scutellaria root, and Ginseng), despite their diverse chemical structures. This distinctive pattern suggests a complex immune response that may involve metabolic transformation of multiple herbal constituents rather than simple structural cross-reactivity [8].

This case must be differentiated from other forms of herbal medicine-associated nephropathy. Unlike aristolochic acid nephropathy (AAN), which causes progressive interstitial fibrosis, tubular atrophy, and cortical shrinkage through direct DNA damage [9, 10], our patient’s condition was characterized by acute inflammation without evidence of direct toxicity. We confirmed with the manufacturer that Saikokaryukotsuboreito does not contain aristolochic acid. Similarly, our case differs from the recently described Beni-koji (red yeast rice) nephropathy [11–13], which presents as Fanconi syndrome and acute kidney injury due to puberulic acid contamination and shows incomplete recovery despite treatment discontinuation. Kidney biopsies in these cases primarily show tubulointerstitial changes, with a recent nationwide survey reporting tubulointerstitial nephritis in 50% and tubular necrosis in 32% of cases [14]. In contrast, our patient’s biopsy lacked tubular necrosis typically seen in Beni-koji nephropathy, and the significant response to glucocorticoid therapy suggested an immune-mediated rather than toxic etiology.

Regarding treatment, while prompt discontinuation of the causative agent is fundamental, glucocorticoid therapy remains beneficial in cases with severe or persistent renal dysfunction. Multiple retrospective studies suggest that early glucocorticoid therapy initiation is associated with improved renal outcomes and reduced progression to chronic kidney disease [15, 16]. In our case, despite initiating prednisolone approximately 50 days after Saikokaryukotsuboreito discontinuation, we observed a favorable response with normalized renal function, except for minor residual tubular acidosis. This outcome suggests that the predominantly inflammatory nature of the lesion, rather than established fibrosis, allowed for effective glucocorticoid responsiveness even with delayed initiation. This finding further indicates that AIN induced by Saikokaryukotsuboreito does not require special consideration and can be approached with the same therapeutic strategies used for conventional drug-induced AIN.

Several limitations should be acknowledged. The sensitivity and specificity of DLST are suboptimal [17], and certain herbs, including Bupleuri Radix (a component of Saikokaryukotsuboreito), may cause false-positive results due to inherent mitogenic activity [18]. In addition, we were unable to test all components of the herbal formulation. Furthermore, serum IgG4 and IgE levels were not measured during the patient’s hospitalization, which might have provided additional insights into the immunological mechanisms involved. Nevertheless, the temporal relationship between drug administration and symptom onset, combined with the consistent positive results across all four tested components, along with the improvement after drug discontinuation and glucocorticoid therapy, strongly supports causality. The fact that CD68-positive cellular infiltration was prominent in the interstitium suggested that although there were no typical giant cells, a foreign body reaction to the suspect drug may have occurred, which may have activated T cells and caused interstitial nephritis.

In conclusion, this case demonstrates that Saikokaryukotsuboreito can cause immune-mediated AIN that responds favorably to glucocorticoid therapy. It highlights that herbal medicines, despite their perceived safety, can induce significant kidney injury through mechanisms similar to conventional pharmaceuticals. Clinicians should include herbal medicines in the differential diagnosis of unexplained AIN and apply standard diagnostic and therapeutic approaches. This case underscores the importance of comprehensive medication history, including traditional remedies, and the value of both renal biopsy and immunological testing in establishing accurate diagnosis and guiding appropriate treatment.

## Supplementary Information

Below is the link to the electronic supplementary material.

Supplementary file1 (PDF 654 KB)
