# Supplementary material for: A case of acute tubulointerstitial nephritis with suspected Saikokaryukotsuboreito involvement responding to glucocorticoid therapy
Source: CEN Case Rep. 2025 Jun 26;14(5):732–9. doi: 10.1007/s13730-025-01012-2 (PMC12457270; doi:10.1007/s13730-025-01012-2)
Supplement: Supplementary file 1 — Supplementary file1 (PDF 654 KB) [file 13730_2025_1012_MOESM1_ESM.pdf]

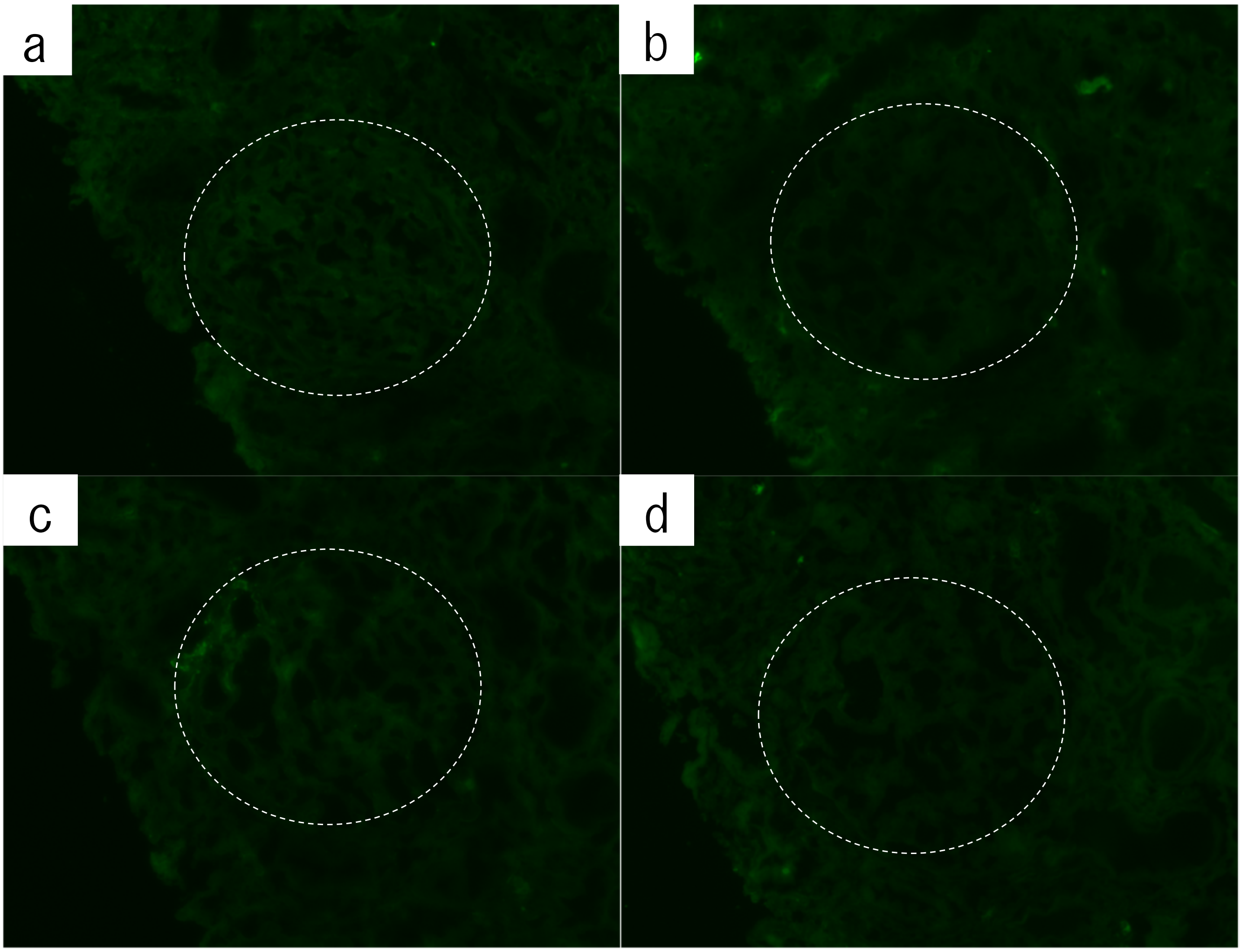


Ishibashi et al. Supplemental Figure.
Immunofluorescence findings of the present case. Anti-IgG (a), IgA (b), C3 (c), C1q (d) stain was negative for a glomerulus (dotted circle), tubular epithelia and tubular basement membrane. Original magnification x200.
